# Age greater than 60 years portends a worse prognosis in patients with papillary thyroid cancer: should there be three age categories for staging?

**DOI:** 10.1186/s12885-018-4181-4

**Published:** 2018-03-22

**Authors:** Rondi M. Kauffmann, J. Blair Hamner, Philip H. G. Ituarte, John H. Yim

**Affiliations:** Division of Surgical Oncology, Department of Surgery, City of Hope National Medical Center, 1500 East Duarte Rd, Duarte, CA 91010-8113 USA

**Keywords:** Papillary thyroid cancer, Thyroid cancer and age, Thyroid cancer staging

## Abstract

**Background:**

Age is an important prognostic factor in papillary thyroid cancer (PTC), with better survival observed in patients < 45 years of age, regardless of stage. Although the impact of increasing age on PTC-related survival is well-known, previous studies have focused on survival relative to age 45 years only. As the number of patients entering their 7th decade of life increases, PTC-related survival in this demographic becomes increasingly important. Survival in patients ≥ 60 years specifically compared to other groups has not previously been examined. We sought to determine whether age ≥ 60 years is an adverse prognostic factor for disease-specific survival and recurrence in patients with PTC.

**Methods:**

The California Cancer Registry database was linked to inpatient and ambulatory patient records from the Office of Statewide Health Planning and Development for the years 2000–2011. This linked database was queried for patients diagnosed with papillary thyroid cancer and treated with surgery. We then identified prognostic factors related to both 5-year and 10-year disease-specific survival and disease-free survival in patients ≤ 45, 45–59, and ≥ 60 years. Multivariable Cox proportional hazard models were created to test the effect of age ≥ 60 on disease-specific and disease-free survival, controlling for clinical, treatment, and demographic factors.

**Results:**

The final cohort included 15,675 patients. Of the group, 46.3% were between 18 and 44 years of age, 33.6% were 45–59 years, and 20.1% were ≥ 60. Univariate analysis showed that compared to other groups, patients ≥ 60 were more likely to be male (*p* < 0.001), present with tumors > 5 cm (*p* < 0.001), more likely to have metastatic disease (*p* < 0.001), less likely to receive radioactive iodine (*p* < 0.001), and more likely to receive external beam radiation therapy (*p* < 0.001). In multivariable Cox proportional hazards models for 5 and 10-year disease-free survival, age ≥ 60 was associated with higher risk of disease at 5 and 10-years (HR 2.3 and 1.9 respectively, *p* < 0.001). Similar results were observed for 5 and 10-year disease-specific survival (HR 38.0 and 30.0 respectively, *p* < 0.001) after controlling for gender, race, co-morbidity, stage, surgical procedure, radioactive iodine, insurance, and hospital volume.

**Conclusions:**

Patients ≥ 60 years of age have worse DSS and DFS after a diagnosis of PTC, across all stages of disease. Given that patients over the age of 45 years have progressively worse survival as they age, these data support having three age groups, 18–44 years of age, 45–59 years, and ≥ 60 as an independent predictor of survival and recurrence to current staging guidelines.

## Background

Thyroid carcinoma is a disease of thyroid tissue, and ranges from well-differentiated to poorly- differentiated. The lifetime risk of developing thyroid carcinoma is approximately 1% in the United States, with the well-differentiated subtype representing most of these cases [1]. As is true of thyroid nodules, thyroid carcinoma is 2–3 times more common in women than men [2]. Overall, the incidence of thyroid carcinoma is increasing, and now represents the 5th most common malignancy in women [2–4].

Age has long been recognized as an important prognostic factor in papillary thyroid cancer (PTC), with better survival observed in patients < 45 years of age, regardless of stage [5–7]. Given that age is one of the most important prognostic factors in PTC, staging systems utilize an age cut-off of 45 years to stratify risk [8].

Recent studies from Asia and Europe have reported that PTC patients older than 60 years tend to have significant shorter survival time compared with younger patients, even after accounting for other predictors [8–10].We sought to determine whether age ≥ 60 years is an adverse prognostic factor for disease-specific survival (DSS) and disease-free survival (DFS) in patients with PTC diagnosed and treated in the United States.

## Methods

This study was approved by both the California Health and Human Services Agency Commission for the Protection of Human Subjects (CPHS), and by the Institutional Review Board of City of Hope Medical Center. Informed consent was waived, since all data is de-identified.

### Data source

The California Cancer Registry (CCR) is maintained by the California Department of Public Health. This statewide population-based cancer surveillance system provides detailed data on all patients diagnosed and/or treated for any primary malignancy (excluding non-melanoma skin cancers) in California. Information includes age, gender, ethnicity, insurance, SES data, tumor site, histology, stage, treatment, and survival (ccrcal.org) [11–13]. The California Office of Statewide Health Planning and Development (OSHPD) database is a state-wide all-payer discharge data set which collects data regarding California’s healthcare infrastructure, outcomes, and facilities [14]. The CCR and OSHPD data sets may be merged to provide comprehensive information about inpatient and outpatient treatments.

### Cohort selection

The CCR data from 2000 to 2011 was queried for patients aged ≥ 18 years, diagnosed with PTC and treated with surgery. PTC was narrowly defined by histologic codes, and excluded micro-papillary pathology (< 10 mm) or cases diagnosed at autopsy or death. These cases were then linked to inpatient and outpatient records from OSHPD for this same time period [15]. Thyroid cancer-related surgical procedures were abstracted using the ICD-9 procedure codes. Cases that had a cervical lymphadenectomy (CLND) (in isolation, as opposed to in combination with thyroidectomy) as their first hospital record were excluded, to avoid including patients with recurrent disease. Patients were considered to have undergone a CLND with their index thyroidectomy if any cervical nodes were recorded as being formally removed with a separate procedure code, including central neck dissection (majority of cases) and lateral neck dissection when applicable. All included patients survived at least 30 days after diagnosis, and only cases whose first or only malignancy was PTC were included. The Deyo modification of the Charlson Comorbidity Index was used to identify patients’ co-morbidities [16].

We then identified prognostic factors related to 5- and 10-year DSS and DFS in patients ≤ 45, 45–59, and ≥ 60 years, including age, gender, race, stage, tumor size, surgery performed, RAI, external beam radiation therapy (EBRT), insurance, hospital volume, and co-morbidities.

Completion thyroidectomy was defined as patients who had a lobectomy followed by re-admission for another lobectomy or total thyroidectomy within 5 months after initial thyroid surgery [15]. Hospital-centered predictors included annual hospital volume, and National Cancer Institute status.

### Statistical analysis

Patient demographics and clinical characteristics were compared between age groups, using Pearson’s chi-squared tests. Multivariable Cox proportional hazard models were created to test the effect of age ≥ 60 on DSS and DFS, controlling for clinical, treatment, and demographic factors. All models excluded cases with unknown/missing stage data. Kaplan-Meier curves were used to analyze survival rates. To evaluate model fit and each model’s ability to predict survivors from non-survivors, we estimated Harrell’s c-statistic, with a c-statistic between 0.70 to < 0.80 having acceptable discrimination [17]. All analyses were performed using the Stata 13, version MP statistical software (StataCorp LP, College Station, TX). The level of statistical significance was set at *p* < 0.05.

## Results

### Patient demographics

A total of 15,675 patients were included, with 22.8% males and 77.2% females. 46.3% were between the ages of 18–44, 33.6% were 45–69, and 20.1% were greater than 60. The majority of patients were non-Hispanic whites (49.2%), with Hispanics (27.3%), and non-Hispanic Asian/Pacific Islanders (16.6%) as the 2nd and 3rd largest groups. Tumor sizes ranged from 1 to ≥ 5 cm. A total of 55.4% of patients had localized disease, 39.2% had regional disease, and 5.3% had metastatic disease. 14.6% of cases were multifocal.

22.6% of patients underwent surgery at a hospital with an annual volume of < 15 thyroidectomies, 28.3% underwent surgery at a hospital with an annual volume of 15 to < 30, 25.2% underwent surgery at a hospital with an annual volume of 30 to < 55, and the remaining 23.9% underwent surgery at a hospital with an annual volume exceeding 55. Insurance coverage for the cohort included Medicaid/indigent (9.5%), Medicare (12.8%), private/HMO (74.9%), VA/Federal (0.9%), and other/unknown (1.9%). Surgery was performed at an NCI-designated hospital in 15.0% of patients.

Surgical treatment included completion thyroidectomy in 5.0% of patients, thyroid lobectomy in 12.4%, total thyroidectomy in 64.2%, and total thyroidectomy with CLND in 18.4% of patients. RAI was administered to 63.0% of patients. EBRT was administered to 1.0% of patients. Most patients were healthy, with 84.2% having no co-morbid conditions, 13.9% having one co-morbidity, and the remaining 1.9% having 2 or more co-morbidities. A total of 74.9% were covered by private health insurance.

### Univariate analysis

Univariate analysis showed that compared to other groups, patients ≥ 60 were more likely to be male, present with tumors ≥ 5 cm, more likely to have metastatic disease, less likely to receive RAI, and more likely to receive EBRT. Patients ≥ 60 years were less likely than all other age groups to undergo completion thyroidectomy, and less likely to undergo total thyroidectomy when compared to patients aged 45–59. Patients in the oldest age group were less likely to undergo CLND (with removal of either central neck nodes or lateral neck nodes) than patients aged 18–44, but more likely to undergo lymphadenectomy than patients in the 45–59 year age group. Patients ≥ 60 were more likely to be covered by Medicare, and less likely to have private/HMO insurance. Co-morbidities were also more common in patients ≥ 60 years, compared to younger groups (Table 1).

**Table 1:** Univariate analysis of patient demographics, clinical characteristics and treatment by age group

	N	Age 18–44 years (%)	Age 45–59 years (%)	Age ≥ 60 years (%)	*p-*value
Gender					
Male	3576	1347 (18.5)	1290 (24.5)	939 (29.8)	*< 0.001*
Female	12,099	5915 (81.5)	3971 (75.5)	2213 (70.2)	
Tumor size					
1 to < 2 cm	6374	2909 (40.1)	2325 (44.2)	1140 (36.2)	*< 0.001*
≥ 2 to < 3 cm	3953	1918 (26.4)	1305 (24.8)	730 (23.2)	
≥ 3 to < 5 cm	3189	1552 (21.4)	955 (18.1)	682 (21.6)	
≥ 5 cm	1288	547 (7.5)	376 (7.2)	365 (11.6)	
Unknown	871	336 (4.6)	300 (5.7)	235 (7.5)	
Stage					
Localized	8686	3922 (54.0)	3.098 (58.9)	1666 (52.9)	*< 0.001*
Regional	6138	3106 (42.8)	1925 (36.6)	1107 (35.1)	
Remote	825	224 (3.1)	228 (4.3)	373 (11.8)	
Unknown	26	10 (0.1)	10 (0.2)	6 (0.2)	
Surgery					
Completion thyroidectomy (TTX)	777	388 (5.3)	272 (5.2)	117 (3.7)	*< 0.001*
Thyroid lobectomy	1950	794 (10.9)	670 (12.7)	486 (15.4)	
Total TTX	10,071	4577 (63.0)	3503 (66.6)	1991 (63.2)	
Total TTX with cervical lymphadenectomy	2877	1503 (20.7)	816 (15.5)	558 (17.7)	
Radioactive iodine					
Yes	9868	4673 (64.3)	3321 (63.1)	1874 (59.4)	*< 0.001*
External beam radiation					
Yes	193	62 (0.8)	51 (1.0)	80 (2.5)	*< 0.001*
Hospital thyroidectomy volume					
< 15 yearly	3545	1632 (22.5)	1180 (22.4)	733 (23.3)	0.081
15 to < 30 yearly	4433	2057 (28.3)	1484 (28.2)	892 (28.3)	
30 to < 55 yearly	3953	1778 (24.5)	1341 (25.5)	834 (26.5)	
55+ yearly	3744	1795 (24.7)	1256 (23.9)	693 (22.0)	
# Of comorbidities					
0	13,200	6591 (90.8)	4363 (82.9)	2243 (71.3)	*< 0.001*
1	2180	630 (8.7)	798 (15.2)	752 (23.9)	
2 +	295	41 (0.60)	100 (1.9)	154 (4.9)	
Race					
Asian/Pacific Islander	2608	1157 (15.9)	864 (16.4)	587 (18.6)	*< 0.001*
Black	527	193 (2.7)	209 (4.0)	125 (4.0)	
Hispanic	4277	2323 (32.0)	1287 (24.5)	667 (21.2)	
White	7707	3316 (45.6)	2727 (51.8)	1664 (52.8)	
Other	556	273 (3.8)	174 (3.3)	109 (3.5)	
Insurance					
Medicaid/indigent	1494	757 (10.4)	539 (10.2)	198 (6.3)	*< 0.001*
Medicare	2002	76 (1.0)	175 (3.3)	1751 (55.5)	
Private/HMO	11,738	6209 (85.5)	4380 (83.2)	1149 (36.4)	
VA/Federal	146	80 (1.1)	46 (0.9)	20 (0.6)	
Other/unknown	295	140 (1.9)	121 (2.3)	34 (1.1)	
Nci hospital					
Yes	2358	1137 (15.7)	773 (14.7)	448 (14.2)	0.114

Italicized *p*-values are statistically significant

### Multivariable Cox proportional hazards model

Predictors of 5-year DSS included advanced disease and larger tumor size (Table 2). There was no association between 5-year DSS and gender, number of co-morbidities, race, type of surgery performed, insurance coverage, or hospital volume. The administration of radioactive iodine was protective (HR 0.50). The hazard ratio for death from PTC at 5 years was 38 times higher in patients ≥ 60 years, compared to patients < 45 years of age (Table 2). Harrell’s c-statistic was 0.9452.

**Table 2:** Multivariable Cox-proportional hazards analysis of predictors of 5-year disease-specific survival

	HR (95% CI)	*p-*value
Stage		
Regional	8.7 (4.8–15.9)	*< 0.001*
Metastatic	40.9 (22.5–74.5)	*< 0.001*
Tumor size		
≥ 2 to < 3 cm	2.0 (1.1–3.6)	*0.021*
≥ 3 to < 5 cm	3.7 (2.2–6.3)	*< 0.001*
≥ 5 cm	6.5 (3.8–11.2)	*< 0.001*
Unknown	5.0 (2.7–9.3)	*< 0.001*
Radioactive iodine		
Yes	0.50 (0.38–0.67)	*< 0.001*
Hospital volume (vs. average < 15 thyroidectomies yearly)		
15 to < 30	1.11 (0.76–1.63)	0.574
30–55	0.81 (0.55–1.21)	0.311
55+	1.16 (0.78–1.72)	0.452
Surgery performed (vs. thyroid lobectomy)		
Completion thyroidectomy	0.23 (0.03–1.73)	0.156
Total thyroidectomy	0.70 (0.47–1.04)	0.078
Total thyroidectomy with cervical lymphadenectomy	1.10 (0.72–1.66)	0.660
Race (vs. Non-Hispanic White)		
Non-Hispanic Black	0.71 (0.26–1.93)	0.501
Hispanic	1.01 (0.73–1.42)	0.915
Asian/Pacific Islander	0.73 (0.49–1.08)	0.120
Other race/Unknown	1.42 (0.76–2.68)	0.274
Age		
45–59	16.43 (6.55–41.18)	*< 0.001*
≥ 60	38.16 (15.08–96.57)	*< 0.001*
Gender		
Female	1.02 (0.76–1.36)	0.890
Comorbidities (vs. none)		
1	0.89 (0.64–1.24)	0.493
2 or more	0.77 (0.39–1.53)	0.457
Insurance (vs. private/hmo)		
Medicare	1.20 (0.83–1.73)	0.334
Medicaid/Indigent	1.40 (0.87–2.25)	0.160
County/Tricare/Military/VA	0.81 (0.11–6.07)	0.839
Not Insured/Other/Unknown	1.70 (0.53–5.45)	0.375

Italicized *p*-values are statistically significant

Predictors of 10-year DSS included advanced disease and larger tumor size (Table 3). There was no association between 10-year DSS and hospital volume, race, co-morbidities, insurance, surgery performed, or gender. The administrative of RAI was protective, with hazard ratio of 0.60. The hazard ratio for death from PTC at 10 years after diagnosis was nearly 30 times higher in patients ≥ 60 years, compared to patients < 45 years of age (Table 3). Harrell’s c-statistic was 0.9328.

**Table 3:** Multivariable Cox-proportional hazards analysis of predictors of 10- year disease-specific survival

	HR (95% CI)	*p-*value
Stage		
Regional	7.3 (4.6–11.6)	*< 0.001*
Metastatic	31.0 (19.5–49.3)	*< 0.001*
Tumor size		
≥ 2 to < 3 cm	1.8 (1.1–2.8)	*0.011*
≥ 3 to < 5 cm	2.8 (1.8–4.2)	*< 0.001*
≥ 5 cm	5.7 (3.7–8.7)	*< 0.001*
Unknown	3.3 (2.0–5.5)	*< 0.001*
Radioactive iodine		
Yes	0.6 (0.5–0.7)	*< 0.001*
Hospital volume (vs. average < 15 thyroidectomies yearly)		
15 to < 30	1.1 (0.8–1.5)	0.488
30–55	0.8 (0.6–1.2)	0.297
55+	1.1 (0.8–1.5)	0.658
Surgery performed (vs. thyroid lobectomy)		
Completion thyroidectomy	0.4 (0.1–1.4)	0.179
Total thyroidectomy	0.8 (0.5–1.1)	0.123
Total thyroidectomy with cervical lymphadenectomy	1.2 (0.8–1.7)	0.305
Race (vs. non-hispanic white)		
Non-Hispanic Black	0.7 (0.3–1.7)	0.491
Hispanic	1.0 (0.8–1.4)	0.713
Asian/Pacific Islander	0.9 (0.6–1.2)	0.543
Other race/Unknown	1.6 (0.9–2.7)	0.093
Age		
45–59	12.2 (6.3–23.6)	*< 0.001*
≥ 60	29.7 (15.2–58.2)	*< 0.001*
Gender		
Female	0.8 (0.6–1.1)	0.157
Comorbidities (vs. none)		
1	0.9 (0.7–1.2)	0.486
2 or more	0.8 (0.4–1.4)	0.429
Insurance (vs. private/hmo)		
Medicare	1.2 (0.9–1.7)	0.191
Medicaid/Indigent	1.4 (0.9–2.0)	0.126
County/Tricare/Military/VA	1.3 (0.3–5.5)	0.719
Not Insured/Other/Unknown	2.0 (0.7–5.6)	0.168

Italicized *p*-values are statistically significant

Predictors of 5-year DFS (Table 4) included advanced disease, number of co-morbidities, and age (HR 2.33 for age ≥ 60 years and 1.3 for age 45–59 years). Increased risk of disease at 5 years was also noted with Hispanic race (HR = 1.5, *p* < 0.001 for Hispanics; was not significant for non-Hispanic blacks, HR = 1.0, *p* = 0.951]), performance of total thyroidectomy, performance of total thyroidectomy with CLND, unknown insurance, and tumor size ≥ 5 cm. Female gender, hospital volume of 30–55 thyroidectomies yearly, and the administration of RAI were associated with a decreased risk of disease at 5 years (Table 4). Harrell’s c-statistic was 0.8316.

**Table 4:** Multivariable Cox-proportional hazards analysis of predictors of 5- year disease-free survival

	HR (95% CI)	*p-*value
Stage		
Regional	3.1 (2.7–3.7)	*< 0.001*
Metastatic	8.2 (6.7–10.0)	*< 0.001*
Tumor size		
≥ 2 to < 3 cm	1.1 (0.9–1.3)	0.351
≥ 3 to < 5 cm	1.1 (0.9–1.3)	0.281
≥ 5 cm	1.9 (1.6–2.4)	*< 0.001*
Unknown	1.2 (0.9–1.5)	0.236
Radioactive iodine		
Yes	0.7 (0.6–0.8)	*< 0.001*
Hospital volume (vs. average < 15 thyroidectomies yearly)		
15 to < 30	1.0 (0.9–1.2)	0.574
30–55	0.7 (0.6–0.9)	*0.001*
55+	1.0 (0.8–1.2)	0.618
Surgery performed (vs. thyroid lobectomy)		
Completion thyroidectomy	0.6 (0.4–1.1)	0.096
Total thyroidectomy	1.3 (1.0–1.6)	*0.014*
Total thyroidectomy with cervical lymphadenectomy	1.8 (1.4–2.2)	*< 0.001*
Race (vs. non-hispanic white)		
Non-Hispanic Black	1.0 (0.7–1.5)	0.951
Hispanic	1.5 (1.3–1.7)	*< 0.001*
Asian/Pacific Islander	1.2 (1.0–1.4)	0.060
Other race/Unknown	1.1 (0.8–1.6)	0.407
Age		
45–59	1.3 (1.1–1.5)	*< 0.001*
≥ 60	2.3 (1.9–2.8)	*< 0.001*
Gender		
Female	0.9 (0.7–1.0)	*0.039*
Comorbidities (vs. none)		
1	1.3 (1.1–1.6)	*< 0.001*
2 or more	1.4 (1.0–2.0)	*0.031*
Insurance (vs. private/hmo)		
Medicare	0.9 (0.8–1.1)	0.601
Medicaid/Indigent	1.1 (0.9–1.3)	0.568
County/Tricare/Military/VA	1.5 (0.8–2.8)	0.199
Not Insured/Other/Unknown	4.5 (3.2–6.2)	*< 0.001*

Italicized *p*-values are statistically significant

Predictors of 10-year DFS (Table 5) included advanced disease, more extensive surgery, Hispanic or Asian/Pacific Islander race, increasing number of co-morbidities, and type of insurance. Age ≥ 60 was associated with increased risk of disease at 10 years. Female gender and administration of RAI were associated with decreased risk of disease at 10 years (Table 5). Hospital volume was not predictive of disease at 10 years except for the category of 30–55 thyroidectomies yearly, which was associated with lower risk. Tumor size increased risk of disease at 10 years only in the ≥ 5 cm group (Table 5).

**Table 5:** Multivariable Cox-proportional hazards analysis of predictors of 10- year disease-free survival

	HR (95% CI)	*p-*value
Stage		
Regional	2.9 (2.6–3.3)	*< 0.001*
Metastatic	6.8 (5.9–7.9)	*< 0.001*
Tumor size		
≥ 2 to < 3 cm	1.1 (0.9–1.2)	0.297
≥ 3 to < 5 cm	1.1 (1.0–1.2)	0.156
≥ 5 cm	1.8 (1.5–2.1)	*< 0.001*
Unknown	1.1 (0.9–1.3)	0.526
Radioactive iodine		
Yes	0.7 (0.7–0.8)	*< 0.001*
Hospital volume (vs. average < 15 thyroidectomies yearly)		
15 to < 30	1.1 (0.9–1.2)	0.242
30–55	0.8 (0.7–0.9)	*0.005*
55+	1.0 (0.9–1.2)	0.806
Surgery performed (vs. thyroid lobectomy)		
Completion thyroidectomy	0.9 (0.6–1.2)	0.419
Total thyroidectomy	1.3 (1.1–1.6)	*< 0.001*
Total thyroidectomy with cervical lymphadenectomy	1.6 (1.4–1.9)	*< 0.001*
Race (vs. non-hispanic white)		
Non-Hispanic Black	1.0 (0.7–1.3)	0.999
Hispanic	1.3 (1.2–1.5)	*< 0.001*
Asian/Pacific Islander	1.2 (1.1–1.4)	*0.003*
Other race/Unknown	1.0 (0.8–1.3)	0.887
Age		
45–59	1.2 (1.1–1.4)	*0.001*
≥ 60	1.9 (1.7–2.2)	*< 0.001*
Gender		
Female	0.8 (0.7–0.9)	*< 0.001*
Comorbidities (vs. none)		
1	1.3 (1.1–1.5)	*< 0.001*
2 or more	1.4 (1.0–1.8)	*0.026*
Insurance (vs. private/hmo)		
Medicare	1.1 (0.9–1.3)	0.270
Medicaid/Indigent	1.2 (1.0–1.4)	*0.016*
County/Tricare/Military/VA	1.9 (1.2–3.0)	*0.003*
Not Insured/Other/Unknown	3.3 (2.4–4.6)	*< 0.001*

Italicized *p*-values are statistically significant

### Survival analysis

**Fig. 1:**
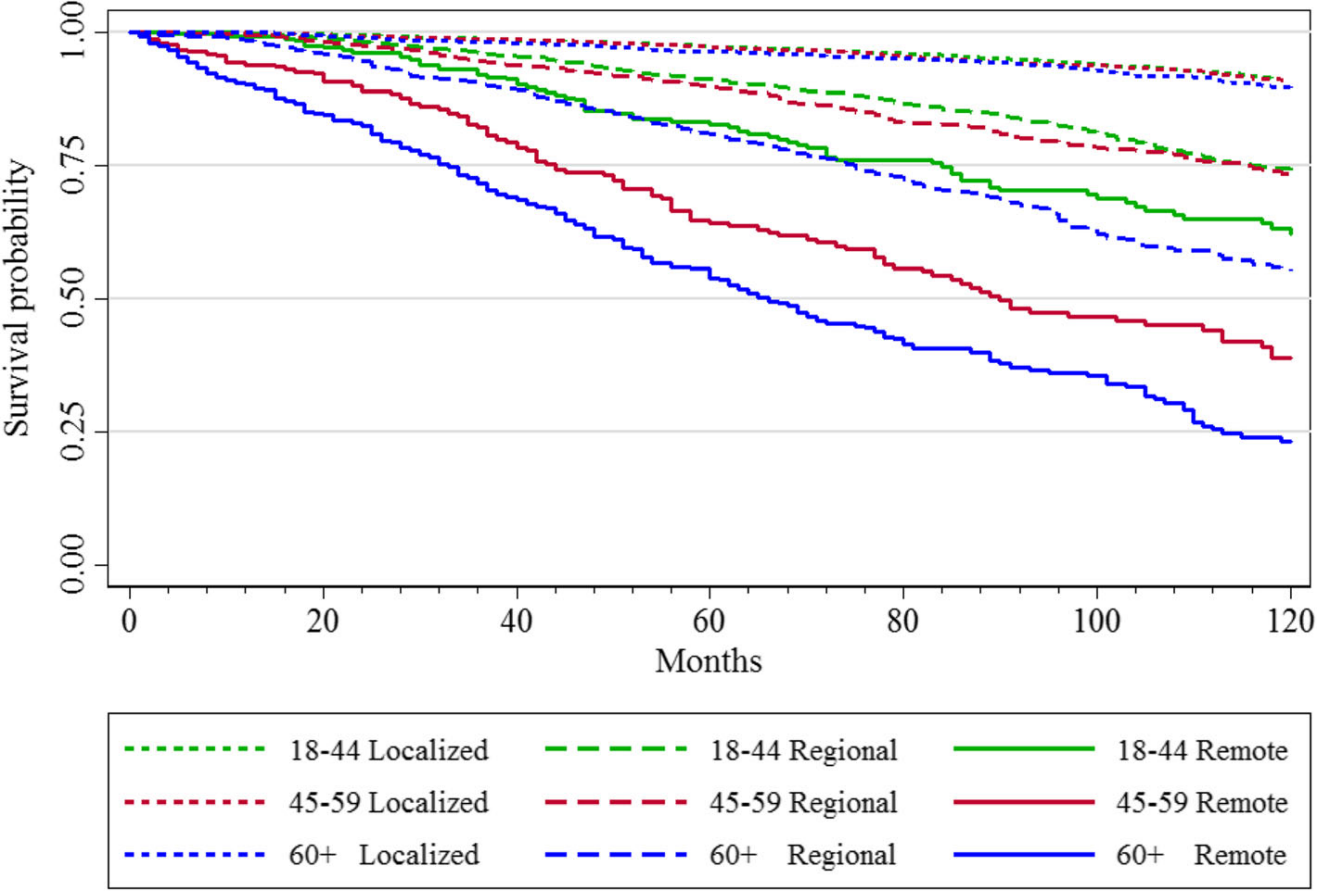
Disease Free Survival at 10 Years by Age Group and Stage

**Fig. 2:**
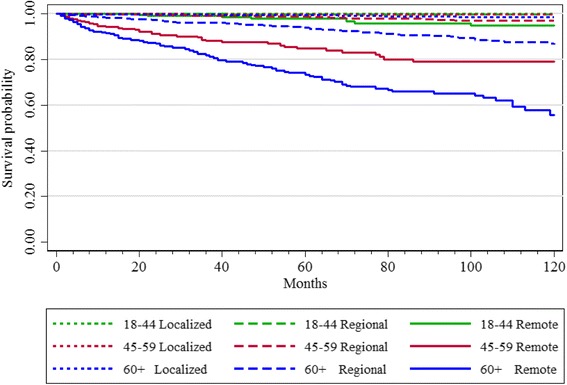
Disease Specific Survival at 10 Years by Age Group and Stage

A total of 1085 deaths from all causes were observed during the study period. 1.5% of patients had died of thyroid cancer by the end of the study period. For thyroid-cancer specific deaths, in patients aged 18–44 years, 14 patients had died, while 87 deaths occurred in patients aged 45–59 years, and 225 deaths occurred in patients aged ≥ 60 years. Deaths were more likely to occur in patients aged ≥ 60 years, across all stages of disease. In univariate Kaplan-Meier curves, the 3-level age-group was significantly associated with DSS and DFS at 10-years, even when data were stratified by cancer stage (*p* < 0.001). Specifically, the log-rank test indicated that the number of observed cancer-specific deaths, or presence of disease at last follow-up among patients age ≥ 60 always exceeded the expected number (*p* < 0.001) (Figs. 1 and 2).

## Discussion

PTC is the most common subtype of thyroid cancer, and age at diagnosis is an important prognostic factor [8, 18, 19]. Age < 45 has historically been used as a cut-off to stratify risk [8]. However, this cut-off makes sense only if one assumes that all patients over the age of 45 have a uniform risk of recurrence and death, whether they are close to the cut-off or far. Recent data has suggested that patients over the age of 45 have progressively worse survival as they age [8–10]. For this reason, another age cut-off of 60 years has been considered. Studies from Europe and Asia have suggested that this second age cut-off may provide additional prognostic information [8–10]. Elisei et al. evaluated a large series of patients with differentiated thyroid cancer from a single institution, analyzing overall survival according to age < 45, 45–60 and > 60 years. They demonstrated that age over 60 years, along with the presence of metastatic disease at diagnosis were the two most important prognostic factors for a shorter survival [20]. Our study has shown similar results, but in a large state database, adding to the body of evidence that age > 60 is an important prognostic factor. Although it is possible that there is a “continuum” of the age related to increased risk of death, the differences in survival from one year of age to the next are so small, as to be indistinguishable. In addition, there is no clear inflection point on ROC curve analysis, and we therefore used the cut-off of age > 60, as reported by other studies.

It is somewhat surprising that only ~ 60% of patients received RAI while ~ 90% of patients received surgeries that would result in near removal of the whole thyroid. In the past, the large majority of patients would receive RAI to ablate the small amount of residual tissue resulting from these procedures. This may reflect the trend toward some limitation of the use of RAI in selected cases and indeed the most recent ATA guidelines published in 2009 do provide recommendations for not treating with RAI [21]. While the overlap of this study with these guidelines was only from 2009 to 2011, it is likely that these trends were occurring prior to the publication of these guidelines. When use of RAI over the study period was examined, factors associated with increased use of RAI included age < 60 years, Hispanic or white race, private/HMO insurance, presence of regional (rather than local or distant) disease, operative procedure of total thyroidectomy with cervical lymphadenectomy (likely correlates with stage of disease), presence of multifocal disease, treatment at a high volume center, and treatment at a non-NCI hospital. There was no association between RAI and gender. However, it is also possible that the finding that ~ 60% of patients received RAI can be explained by a coding error. In our cohort, 10% of patients receiving RAI were coded as having undergone lobectomy. Patients undergoing lobectomy alone would not be expected to receive RAI, and it is therefore possible that these patients’ procedures were mis-coded as “lobectomy”, when they had actually undergone a completion lobectomy. Such coding errors are known to occur in large databases [22, 23]. As expected, the use of EBRT was very limited, with only 1.0% of patients receiving this therapy. Although EBRT is used more liberally in parts of Asia [18], it is used only very selectively in the United States.

Our analysis suggests that RAI is associated with significantly improved DSS for the entire cohort (all ages). In light of the 2009 ATA guidelines, RAI would have been used in higher-risk patients with PTC, but not in low-risk patients. Although our 12-year cohort included 9 years of patients diagnosed before publication of the 2009 ATA guidelines, it is likely that clinicians were using clinical judgement in identifying high-risk patients, for whom RAI would be beneficial, even before this recommendation was reflected in national guidelines. It is therefore not surprising that RAI is associated with improved DFS and DSS, given the use of RAI only in those with high risk disease [21].

It is interesting that univariate analysis showed that patients ≥ 60 were more likely to be male, present with tumors ≥ 5 cm, and have metastatic disease at presentation. Because PTC is more common in females, the larger-than-expected percentage of elderly men with PTC in our cohort is somewhat surprising. PTC in males tends to be more aggressive, so our findings that these individuals also tend to have larger tumors and metastatic disease at diagnosis is entirely consistent with accepted principles that outcomes are better in women than men [21]. However, a recent publication evaluated the relationships between gender, age, and PTC outcomes, and found that while younger women tend to have better survival than men, outcomes are similar in older patients [24].This suggests that older age may modify the effect of gender on outcomes from PTC, and that an older age cut-off may be warranted [24].

Additionally, older patients were less like to undergo total or completion thyroidectomy or RAI, and were more likely to receive EBRT. EBRT is indicated primarily for adjuvant use in high-risk patients, or those with unresectable cancer. The higher frequency of EBRT in older patients in our cohort may represent a tendency for surgeons to limit extent of surgical treatment in favor of a lesser surgery in combination with radiation, in patients with co-morbid conditions [25].

We have also shown that DSS and DFS are decreased in patients ≥ 60 years, after controlling for gender, race, co-morbidities, stage, tumor size, extent of surgical treatment, RAI, insurance status, and hospital volume. Similar studies from Asia and Europe have shown that patients ≥ 60 years fare worse than those over 45 years, but younger than 60 years. Our study confirms the importance of age ≥ 60 years on PTC-related survival in an American cohort. This suggests that the biology of disease in elderly patients is inherently more aggressive than disease in younger individuals. Whether this is due to the accumulation of exposures over a lifetime (ionizing radiation from the environment, other environmental toxins, or some other risk factor) is unclear from this analysis. Additionally studies are needed to investigate the underlying causes of the decreased survival in elderly patients with PTC.

The recent publication of the 8th edition of the TNM staging guidelines for thyroid carcinoma changed the “low risk” age cut-off rom 45 years to 55 years. This resulted in many patients being reclassified with low risk disease [26]. Our data would suggest that another “high risk” cut-off of greater than 60 years should be considered.

This study has noteworthy limitations. While the linked data set includes extensive demographic and clinical information on more than 3.4 million cases of cancer diagnosed in California, potentially important covariates were unavailable. Although history of radiation exposure is associated with increased the risk of thyroid malignancy, and with more aggressive forms of disease [27], this information is not collected by CCR or OSHPD. Neither database collects information on previous radiation, and therefore, this was not controlled for in our analysis. Additionally, although insurance status was not a significant predictor of DSS, other socioeconomic factors, such as rural vs. urban living, distance to health care facility, or income may influence timing of seeking medical attention, or availability of endocrinologists and endocrine surgeons. Furthermore, while the data set captures hospital volume, it does not include reliable information on individual surgeon volume, which is known to affect patient outcomes [28]. Coding errors also occur in large, population-based databases, and these would be expected to adversely affect the reliability of the data in regards to RAI and surgical procedure performed, in particular [22, 23]. Patients coded as “lobectomy” may have actually undergone “completion thyroidectomy”, but the veracity of the coded procedure cannot be verified in this dataset, as it is de-identified. While single-institution studies are not subject to issues of coding, as occur in population-based datasets, they also cannot achieve accumulation of similarly large of numbers of patients. Although some studies have suggested that coding errors may occur in up to 10% of records, with sufficient numbers of subjects, this is unlikely to affect study results. However, we acknowledge that this remains an important limitation of studies employing large population-based datasets (such as this one) until better methods of data entry and extraction are available, or until records can be retrospectively verified. Finally, we acknowledge that some of the decrease in DSS and DFS observed in patients ≥ 60 years may be due in part to delay in diagnosis in elderly patients. Additional studies are warranted to further examine this possibility.

The strengths of our study include that information was obtained from a large, prospectively-maintained population-based data set, including all cases of PTC diagnosed and treated in California from 2000 to 2011. Additionally, the linkage between the CCR and the OSHPD data sets, allowed us to control for number of co-morbidities and treatments rendered.

## Conclusions

Patients ≥ 60 years of age have worse DSS and DFS after a diagnosis of PTC, across all stages of disease. Given that patients over the age of 45 years have progressively worse survival as they age, these data support having three age groups, 18–44 years of age, 45–59 years, and ≥ 60 as an independent predictor of survival and recurrence to current staging guidelines.
